# An optimized protocol for generation and analysis of Ion Proton sequencing reads for RNA-Seq

**DOI:** 10.1186/s12864-016-2745-8

**Published:** 2016-05-26

**Authors:** Yongxian Yuan, Huaiqian Xu, Ross Ka-Kit Leung

**Affiliations:** BGI-tech, BGI-Shenzhen, Shenzhen, 518083 Guangdong, China; BGI-tech, BGI-Wuhan, Wuhan, 430075 Hubei, China; School of Public Health, The University of Hong Kong, Hong Kong, China; Stanley Ho Centre for Emerging Infectious Diseases, The Chinese University of Hong Kong, Hong Kong, China

**Keywords:** Ion Proton, RNA-Seq, Transcriptome, Sequencing length, Sequencing quality

## Abstract

**Background:**

Previous studies compared running cost, time and other performance measures of popular sequencing platforms. However, comprehensive assessment of library construction and analysis protocols for Proton sequencing platform remains unexplored. Unlike Illumina sequencing platforms, Proton reads are heterogeneous in length and quality. When sequencing data from different platforms are combined, this can result in reads with various read length. Whether the performance of the commonly used software for handling such kind of data is satisfactory is unknown.

**Results:**

By using universal human reference RNA as the initial material, RNaseIII and chemical fragmentation methods in library construction showed similar result in gene and junction discovery number and expression level estimated accuracy. In contrast, sequencing quality, read length and the choice of software affected mapping rate to a much larger extent. Unspliced aligner TMAP attained the highest mapping rate (97.27 % to genome, 86.46 % to transcriptome), though 47.83 % of mapped reads were clipped. Long reads could paradoxically reduce mapping in junctions. With reference annotation guide, the mapping rate of TopHat2 significantly increased from 75.79 to 92.09 %, especially for long (>150 bp) reads. Sailfish, a k-mer based gene expression quantifier attained highly consistent results with that of TaqMan array and highest sensitivity.

**Conclusion:**

We provided for the first time, the reference statistics of library preparation methods, gene detection and quantification and junction discovery for RNA-Seq by the Ion Proton platform. Chemical fragmentation performed equally well with the enzyme-based one. The optimal Ion Proton sequencing options and analysis software have been evaluated.

**Electronic supplementary material:**

The online version of this article (doi:10.1186/s12864-016-2745-8) contains supplementary material, which is available to authorized users.

## Background

High-throughput RNA sequencing (RNA-Seq) is a powerful tool for transcriptome research of gene expression quantification, alternative splicing detection, gene regulation and single nucleotide polymorphisms (SNPs) discoveries [1–7]. Since the rapid development of sequencing technology in the last decade, several sequencing platforms such as Roche 454, Illumina HiSeq, Life Technologies SOLiD, Personal Genome Machine (PGM) and Proton and Pacific Biosciences RS have been released, which facilitate large-scale transcriptome studies [8–10].

Previous studies conducted by the Association of Biomolecular Resource Facilities (ABRF) and the Sequencing Quality Control Consortium (SEQC) reported high intra- and inter-platform concordance in RNA-Seq among HiSeq, PGM and Proton, SOLiD, 454 and PacBio RS [11, 12]. However, bias and artifacts that can be introduced during different stages of library construction, such as RNA fragmentation, reverse transcription, phosphorylation, and adaptor ligation [13–16], were not studied in details for their possible consequences for the Ion Proton platform. Moreover, many popular RNA-Seq analysis tools were developed based on HiSeq data featured with high accuracy and equal read length, whilst sequencing data generated from Proton and PacBio RS are prone to indels and of variable read length [11, 17]. Whether the analysis strategies and software for HiSeq data can be applied directly to the Ion Proton data has not been evaluated. For examples, the ABRF study [11] showed that the performance of GMAP [18] could achieve at about 90 % mapping rate but for STAR [19] only 60 % for PacBIO sequencing reads. The lower mapping rate by STAR was also observed for Proton data (50 %), when compared with that by TMAP (80 %). We also reported that care has to be taken for the detection of minor variants from sequencing errors on sequencing data generated by PGM [20].

Several studies demonstrated the potential of semiconductor sequencing applications on bacterial genome assembly, target region sequencing and RNA-Seq in a cost-effective way with a rapid turnaround [21–23]. At present more than one-third of RNA-Seq conducted by Beijing Genome Institute is for gene expression quantification, where accuracy at individual position is not the major concern. So is the case in single genome assembly with sufficient coverage. In 2012, Life Technologies released the next-generation semiconductor sequencer Proton, with much higher throughput (at gigabases) than PGM (at megabases), which expands semiconductor sequencing to the applications to whole exome and transcriptome sequencing [17, 24]. Ease of operation and maintenance and short run time of both the PGM (and applicable to Proton) platform attracted much attention [12, 23], because these are crucial factors in pathogen detection in outbreak investigations [23] and clinical applications such as disease biomarker detection, cancer diagnostics and therapeutics and prenatal diagnosis [25, 26].

Library preparation is the first step before sequencing. First of all, long contiguous RNAs have to be fragmented and enzyme digestion is a common option. RNaseIII is a ribonuclease that can digest eukaryotic single strand RNAs (ssRNAs) at specific sites, or recognize and cleave double stranded RNA (dsRNA). During eukaryotic dsRNA metabolism, RNaseIII cleavage usually generates both short and long fragments, and is generally considered to be a random cutter [27, 28]. Therefore it has been successfully applied in NGS RNA-Seq library construction [29]. However, some studies also pointed out that bias can be introduced by RNaseIII fragmentation on SOLiD sequencing platform, as RNaseIII has preferred cutting sites [13]. How these biases are manifested in variable-length sequence generation platforms remains to be investigated. To identify suitable protocols and software for processing Proton data in RNA-Seq applications, we constructed universal human reference RNA (UHRR) libraries by RNaseIII and chemical fragmentation with different initial amounts of RNA and library insertion sizes, and compared the performance of a repertoire of software originally developed for analyzing HiSeq data on Proton sequencing data.

Reliable alignment is usually the pre-requisite for analyses like gene expression quantification, alternative splicing detection and SNP calling [30]. Aligning RNA-Seq data to a eukaryotic genome is more challenging than to its transcriptome, because many genes exhibit multiple exon-intron architecture in a genome, while reads sequenced from mature mRNA transcripts are intron-free. Moreover, in mammalian genomes, introns can span a very wide range of lengths, typically from 50 to 100,000 bases. Since longer reads are more likely to span (more) exons, programs were developed to deal with the reads across exon-exon junction sites when aligning RNA-Seq data to a reference genome [19, 31]. Software and methods were also developed to accommodate the features of sequencing reads generated from different sequencing platforms, such as short read aligner Bowtie2 [32] and BWA [33] for SOLiD and HiSeq sequencing data, GS Reference Mapper(GSRM) (http://www.454.com/products/analysis-software/) for 454 sequencing data, TMAP (https://github.com/iontorrent/TMAP) for Ion Proton/PGM sequencing data, and BWA-SW [34] and GMAP [18] for long read alignment.

Gene detection and expression quantification have long been mature and important applications in RNA-Seq, a number of methods and software for estimating genes and transcripts abundance have been released over the past years. To quantify gene expression level, the first step is to find out how many reads assigned to a certain gene or transcript. Based on Fragments Per Kilobase of transcript per Million mapped reads (FPKM) [2], Reads Per Kilobase of transcript per Million mapped reads (RPKM) [5] or Transcripts Per Million (TPM) [35], there are two major types of gene quantification method. One is alignment-based, calculating from transcriptome alignment results such as RSEM [36], BitSeq [37], eXpress [38], IsoEM and its variation tailor-made for Ion Torrent Data MaLTA-IsoEM [39, 40] or genome alignment results such as Cufflinks/Cuffdiff [41], HTseq [42] and MISO [43]; it is also important to differentiate count-based methods (e.g., HTseq [42]) and methods that first estimate transcripts frequencies by which gene expression levels are estimated (e.g., RSEM [36], Cufflinks [41]); the other is alignment-independent, for example, Sailfish [44]. To evaluate the performance of Proton in gene expression quantification, we selected several combinations of alignment and quantification software for both transcriptome and genome mapping analyses.

## Result

### Library preparation and sequencing statistics

**Fig. 1:**
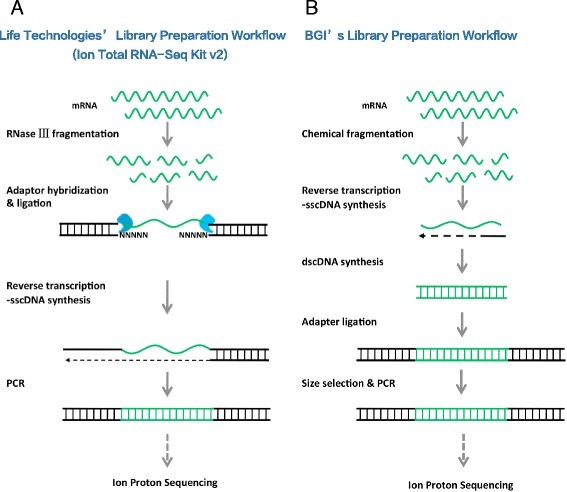
Ion Proton RNA-Seq library preparation workflow. **a** Life Technologies’ protocol: mRNAs were fragmented by RNaseIII using the Ion Total RNA-Seq Kit V2, only the sense RNA strands (mRNA) were sequenced. **b** BGI’s protocol: based on chemical (heat) fragmentation. Additional size selection steps were introduced after adaptor ligation

Two libraries were constructed by RNaseIII fragmentation according to Ion Total RNA-Seq Kit v2 specifications (Life Technologies, Fig. 1a), the other nine were by chemical fragmentation according to the BGI protocol (Fig. 1b), yielding a total of more than 204 million reads. Along with two additional HiSeq RNA-Seq libraries sequenced on HiSeq 2000, the detailed information of every library is presented in Table 1 and Additional file 1: Figure S1 and Additional file 2: Figure S2. The sequencing data are deposited in Sequence Read Archive with project ID SRP064015. The Ion Proton sequencing reads had variable length, peaking at around 150 ~ 200 bp and ranging from 30 to over 300 bp (Additional file 1: Figure S1). The median and interquartile range (IQR) of read length, proportions of Q10, Q20 and Q30 reads were (140.0; 18.5) and (98.82 %, 72.14 %, 1.730; 0.08 %, 2.81 %, 0.35 %) respectively.

**Table 1:** Sequencing platforms, library preparation, and sequencing results

Sequencer	Fragmentation methods	Initial RNA(μg)	Library name	Library insert size(bp)	Mean read length(bp)	Raw Q10	Raw Q20	Raw Q30	Total reads	Total base
Ion Proton	Chemical	2	ProC_1	196	153	98.69 %	70.85 %	1.55 %	19,448,438	2,975,809,154
		2	ProC_2	179	138	98.70 %	69.81 %	1.34 %	25,262,605	3,505,548,278
			ProC_3	200	155	98.82 %	72.14 %	1.80 %	8,139,486	1,263,964,775
		2	ProC_4	166	136	98.80 %	72.31 %	1.79 %	23,866,259	3,267,082,715
			ProC_5	196	155	98.82 %	73.90 %	1.88 %	15,461,415	2,408,389,343
		0.2	ProC_6	175	137	98.74 %	69.79 %	1.35 %	22,673,571	3,123,633,358
			ProC_7	215	158	98.75 %	70.67 %	1.43 %	13,098,194	2,076,154,568
		0.2	ProC_8	180	140	98.82 %	71.57 %	1.61 %	23,142,854	3,251,986,105
			ProC_9	199	160	98.85 %	73.24 %	1.73 %	12,219,286	1,955,402,424
	RNaseIII	2	ProR_1	89	94	99.25 %	83.05 %	4.04 %	16,024,785	1,519,618,514
		2	ProR_2	91	92	99.17 %	81.95 %	3.87 %	25,434,638	2,364,470,896
HiSeq 2000	Chemical	2	HiSeq_1	193	90;90	98.11 %; 95.11 %	97.03 %; 93.73 %	91.86 %; 88.43 %	60,313,246	10,856,384,280
		2	HiSeq_2	187	90;90	97.72 %; 94.53 %	96.59 %; 93.06 %	91.51 %; 87.58 %	56,610,071	10,189,812,780

### Alignment

**Fig. 2:**
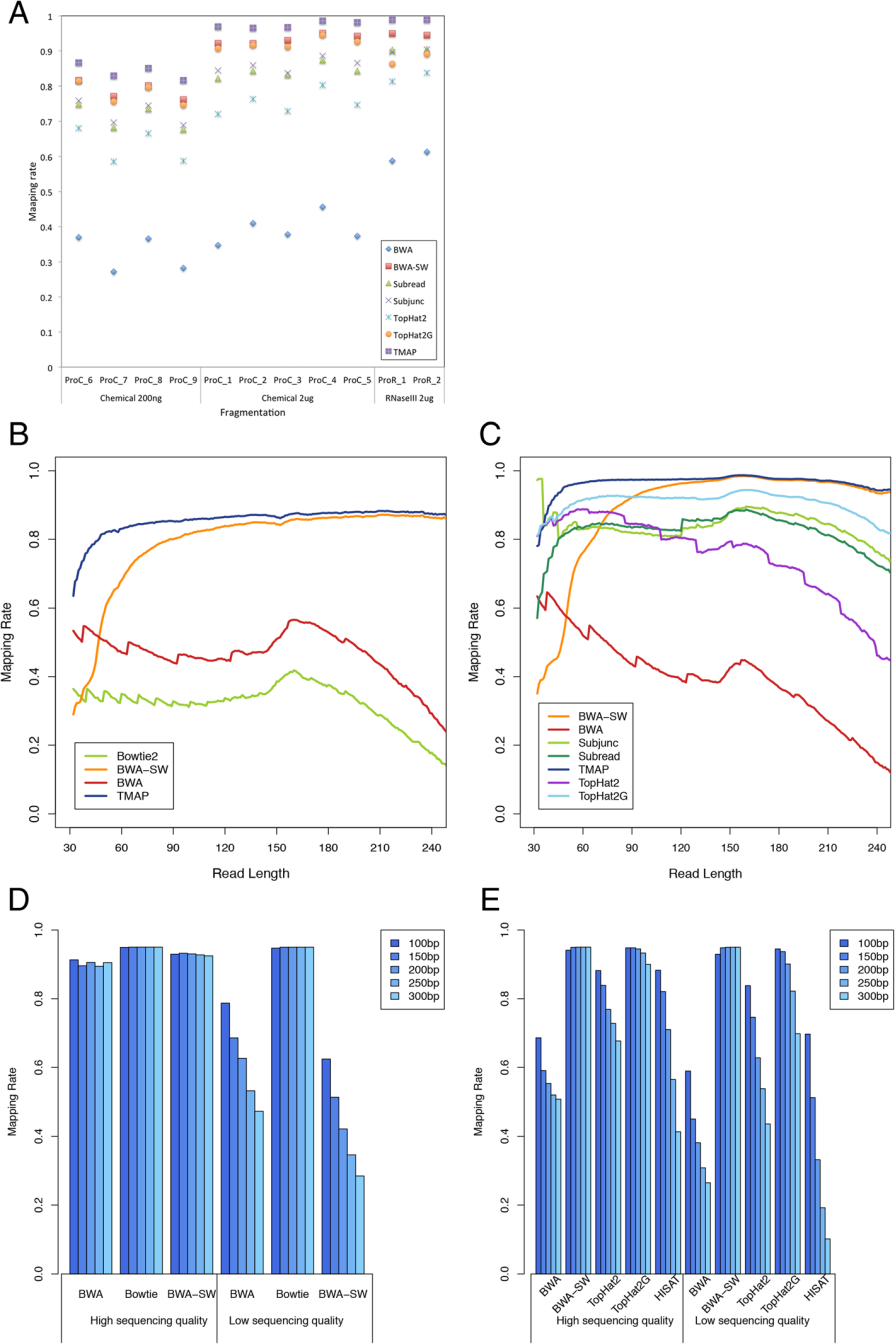
Comparison of mapping rates among **a** libraries by different amount of RNA, software and fragmentation methods, **b** unspliced aligners to the human transcriptome by read length, **c** unspliced and splice-aware aligners to the human genome by read length, **d** unspliced aligners in aligning simulated data to the human transcriptome by read length and sequencing quality, **e** unspliced and splice-aware aligners in aligning simulated data to the human genome by read length and sequencing quality

The mapping rates among Ion Proton sequencing reads by different library preparation conditions and programs are depicted in Fig. 2a. The mapping rates for little starting material (200 ng) were expectedly lower than those for 2ug. The average performance of aligners in mapping Ion Proton reads prepared by all the 2ug libraries is further summarized in Table 2. The proportion of proton reads that could be mapped to the reference human transcriptome was small by BWA (49.87 %) and Bowtie2 (31.04 %), in contrast to that by BWA-SW (82.28 %) and TMAP (86.46 %). The mapping rates by BWA and Bowtie2 declined significantly when read lengths were larger than 150 bp (Fig. 2b). TMAP attained the highest mapping rates and the rate did not decrease with increasing read length, so did BWA-SW. Nevertheless, the performance of BWA-SW was worse than that of TMAP for reads shorter than 120 bp (Fig. 2b). The difference in mapping rates to the human transcriptome and genome between BWA and BWA-SW could be as high as up to 60 and 80 % respectively.

**Table 2:** Average performance of aligners in mapping Ion Proton reads prepared by all the 2ug libraries (see Table 1 for details) to reference human genome and transcriptome

Aligner type	Software	Percentage of reads aligned to reference human	
		Genome	Transcriptome
Unspliced	Bowtie2	–	31.04 %
	BWA	39.90 %	49.87 %
	BWA-SW	93.25 %	82.28 %
	TMAP	97.27 %	86.46 %
	Subread	84.44 %	ND*
Splice-aware	Subjunc	85.99 %	ND
	TopHat2	75.70 %	ND
	TopHat2G	92.09 %	ND

*Not done

Likewise, the performance of BWA also deteriorated for longer read length and so did TopHat2. The other aligners were less affected. When reference junction annotation was allowed to guide the alignment by TopHat2 (TopHat2G), the mapping rate elevated by as high as 40 % for long reads (Fig. 2c).

To determine whether the low alignment rates with long reads in general (Fig. 2) were due to read length or sequencing errors (or read quality), we simulated RNA-Seq data with either high or low sequencing quality (for details see Method) and of different length. For the alignment of high quality reads to reference transcriptome, BWA, Bowtie2 and BWA-SW all manifested more or less the same mapping rates by different read lengths. In contrast, low quality reads decreased mapping rates of BWA and Bowtie2 with increasing read length, which suggests that the low mapping rate with long reads can be caused by the accumulation of sequencing errors (Fig. 2d). Similar phenomenon was observed in genome alignment. The good performers BWA-SW and TopHat2G were insensitive to read quality and length. Moreover, since TopHat2G far outperformed TopHat by the same length, junction alignment complexity was also likely responsible for reduced mapping rates (Fig. 2e).

### Gene detection and expression quantification

**Fig. 3:**
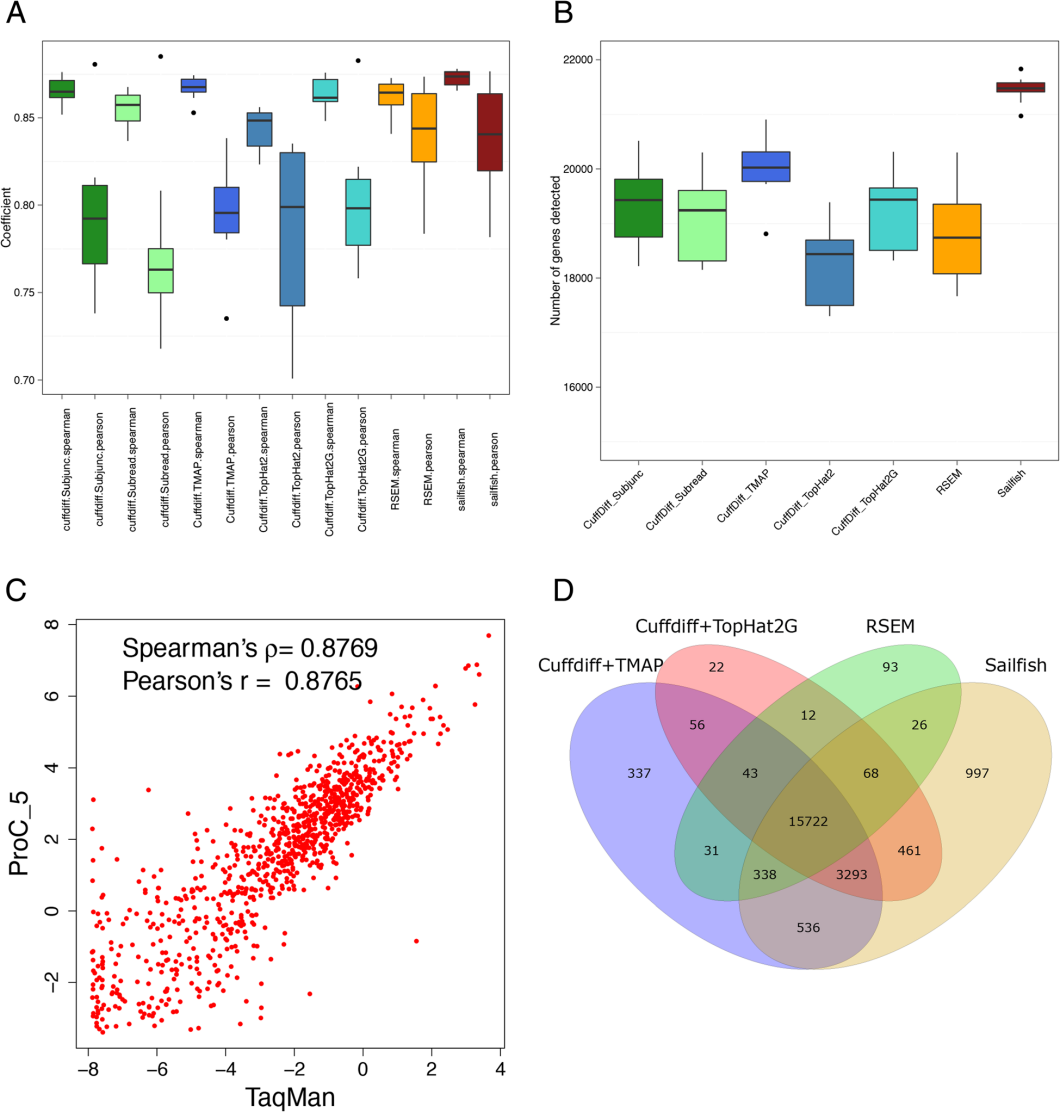
Comparison of Cuffdiff based methods (Cuffdiff + Subjunc, Cuffdiff + Subread, Cuffdiff + TMAP, Cuffdiff + TopHat2, Cuffdiff + TopHat2G), RSEM and Sailfish among **a** robustness in gene expression quantification accuracy, in which all methods yielded consistent results with MAQC-I TaqMan, such that high Spearman correlation coefficients were observed in general, while Pearson correlation coefficients of RSEM and Sailfish were higher and more stable than Cuffdiff based methods, **b** the number of detected genes. Sailfish detected more gene than any other methods. **c** Good concordances were observed between relative expression measures from the MAQC-I TaqMan and Proton platforms, **d** consistency in gene detection. A large proportion of genes were commonly detected by Cuffdiff + TMAP, Cuffdiff + TopHat2G, RSEM and Sailfish, although Sailfish detected more unique genes than the others

All methods showed high consistency with the TaqMan array results obtained from MAQC I project in expression quantification in general (median correlation coefficients > 0.80) but the dispersion of Pearson correlation coefficients were higher (interquartile range = 0.066) than Spearman’s (interquartile range = 0.015) (Fig. 3a). Quantification by transcriptome-based RSEM and alignment-free Sailfish was in general higher and more robust than genome-based Cuffdiff-based methods under various conditions, consisting of fragmentation methods, sequencing depth, initial RNA input and insert size (Fig. 3a).

Sailfish was more sensitive in gene detection than other software, while RSEM detected much fewer genes, most of which can also be found in other gene quantification strategies (Fig. 3b and d). Cuffdiff-based strategies manifested variable performance in mapping rate and correlation with TaqMan array results (Additional file 3: Figure S3C and D). The higher the mapping rate (Table 2), the more was correlated with TaqMan array results (Fig. 3a), and could more genes be detected (Fig. 3b).

### Junction discovery

Compared to Microarray, a unique application of RNA-Seq is to detect alternative splicing events and discover novel isoforms. The very first step of these analyses involves junction discovery. To estimate the performance of the Proton platform in junction discovery, we compared the results obtained from TopHat2, TopHat2G and Subjunc in the numbers of total and annotated detected exon-exon junctions.

**Fig. 4:**
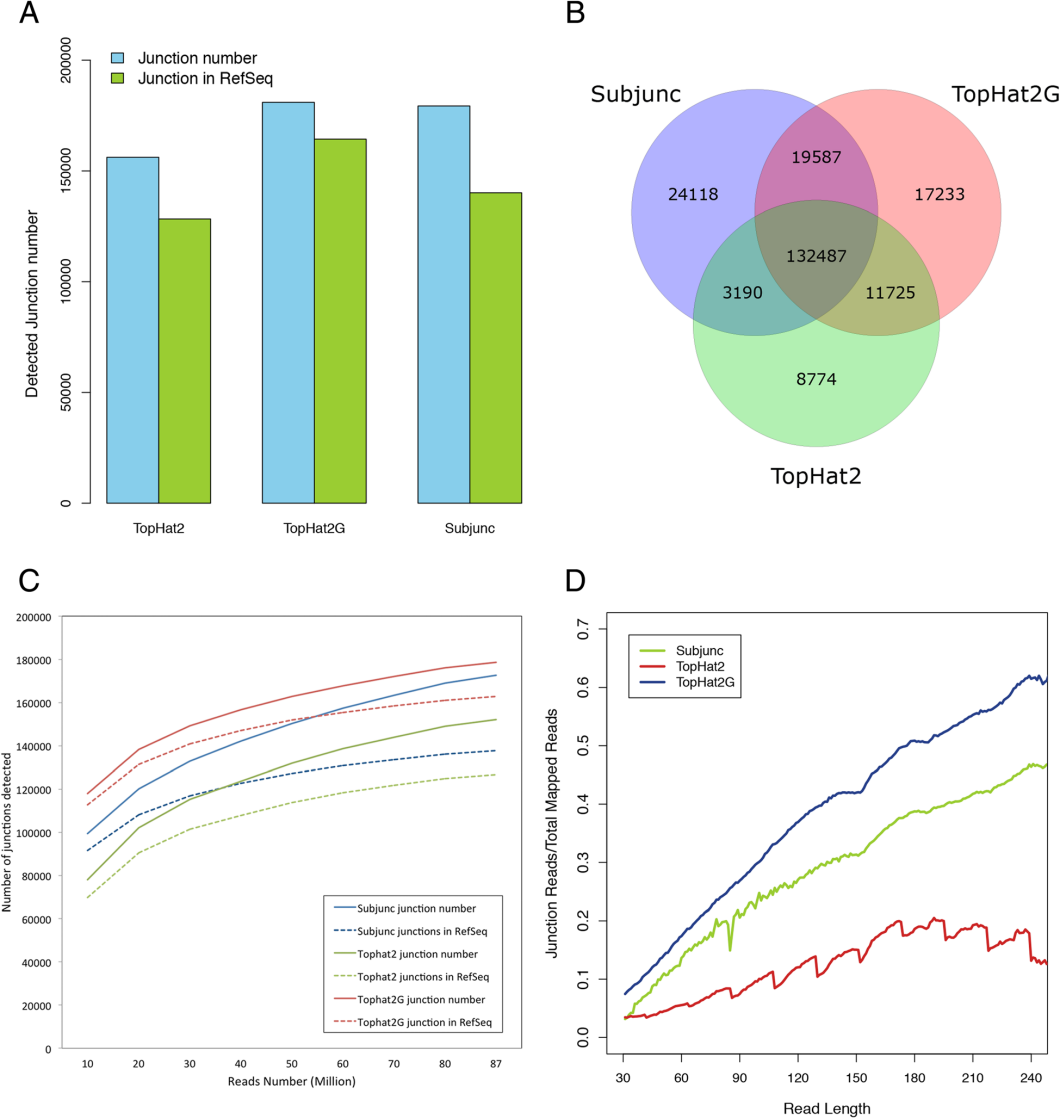
Comparison of Subjunc, TopHat2 and TopHat2G among **a** total detected junction number and the number of junctions in RefSeq (known junctions), **b** junction detection. Over 130,000 junctions were detected by all the three methods, yet a large number of junctions were uniquely identified by individual method, **c** sequencing saturation in junction discovery. As sequencing depth increasing, Subjunc detected more novel junctions (total junctions minus junctions in RefSeq) than TopHat2 and TopHat2G, **d** efficiency of junction reads alignment. *X axis* is the read length; *y axis* is the proportion of junction reads in all mapped reads (junction reads number/total mapped reads number). TopHat2G is more efficient in aligning junction reads compared to Subjunc and TopHat2, especially for long reads

All programs detected more than 150,000 junctions covered by 40 million reads (ProC_1, ProC_2, ProC_3, ProC_4 and ProC_5 in Table 1) (Fig. 4a), with over 130,000 junctions were commonly detected by all the three methods. However, there remained a large number of junctions detected by individual methods (Fig. 4b). Subjunc detected more junctions than TopHat2, however when reference junction annotation (reference gtf) was allowed to guide alignment for TopHat2 (TopHat2G), the number of detected junctions significantly increased, especially for the annotated junctions (Fig. 4a and c). As the sequencing depth increased, Subjunc detected more novel junctions (20.29 % novel junctions/total junctions) than TopHat2 (16.74 %) and TopHat2G (8.92 %) (Fig. 4c). The proportion of junction reads in total aligned reads increased along with read length in general and TopHat2G was more efficient in aligning junction reads than Subjunc and TopHat2, especially for long reads (Fig. 4d).

### Library preparation based on chemical and RNaseIII fragmentation method

**Fig. 5:**
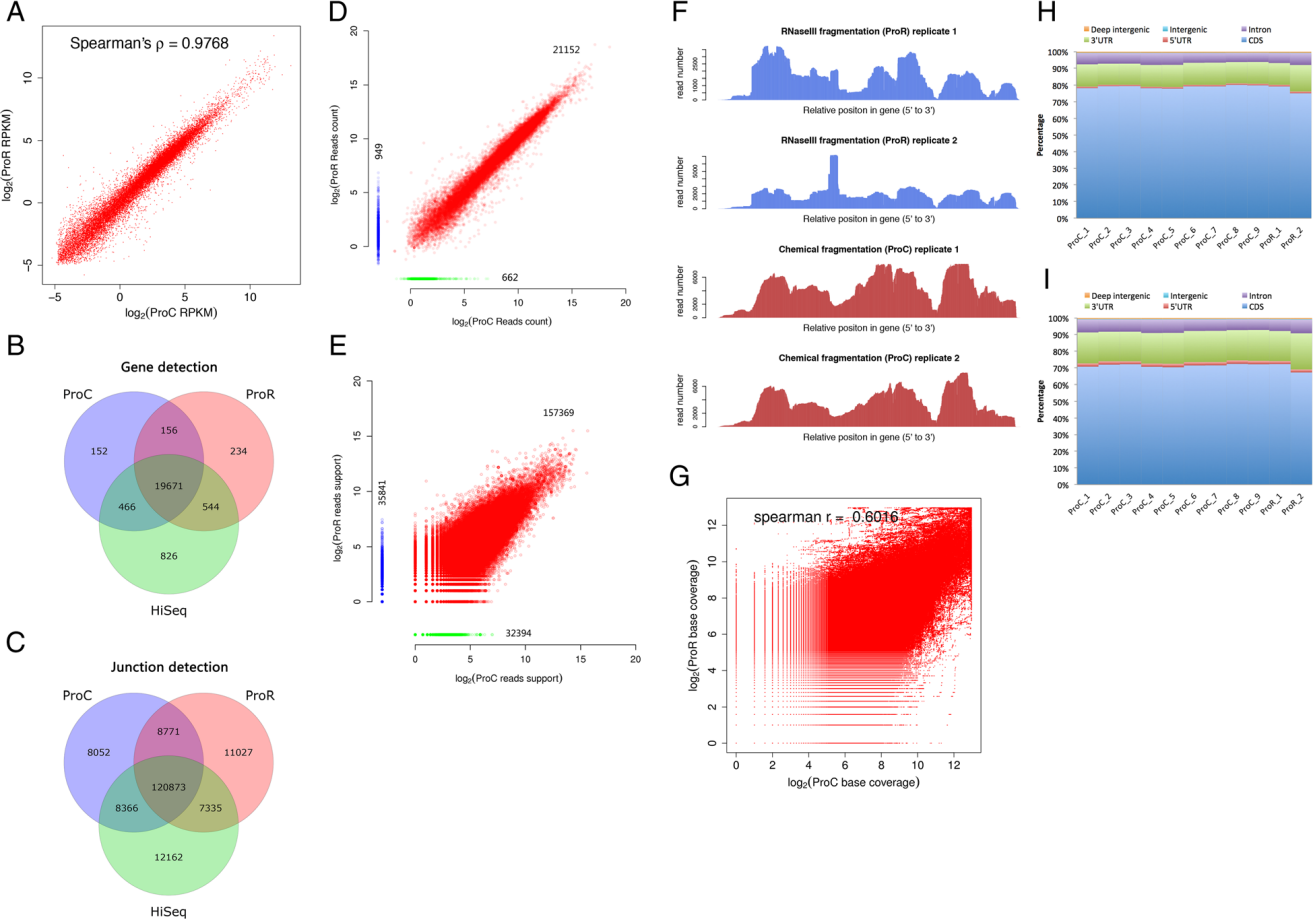
Comparison of library preparation methods among **a** consistency of gene expression quantification. RNaseIII (ProR) and chemical fragmentation (ProC) methods showed high consistency in gene expression quantification. **b**-**c** Genes and junctions detected. Large proportions of genes and junctions were commonly detected by all the three libraries. **d**-**e** Comparison of gene and junction expression level by ProC and ProR fragmentation method. The number of reads mapping onto gene and junction regions are plotted as dots. **d** 949 genes were only detected by ProC but not by ProR, while 662 genes were only detected by ProR. **e** 35841 junctions were only detected by ProC but not by ProR, while 32394 junctions were only detected by ProR, **f** coverage of a house-keeping gene beta actin (ACTB). Similar coverage patterns were observed between technical replicates, but not between library preparation protocols. **g** Comparison in consistency between ProC and ProR fragmentation methods by base coverage, the percentage of reads mapped to various gene sequence categories according to GENCODE v24 **h** Comprehensive and **i** Basic gene annotation

In this study, we constructed RNA-Seq libraries based on two protocols: one with RNaseIII fragmentation (ProR) with Ion Total RNA-Seq kit v2, the other with chemical fragmentation (ProC) according to BGI’s protocol. The two methods were consistent in gene expression quantification (Spearman correlation >0.97) (Fig. 5a) and detection (96.6 % overlap) (Fig. 5b), and junction discovery (88.2 % overlap) (Fig. 5c).

We also compared the read counts mapped to each gene from the libraries constructed with Ion Total RNA-Seq kit v2 protocol (ProR) and BGI protocol (ProC). Some genes and junctions were identified with high read depth according to one method but not the other (Fig. 5d and e). The effect was possibly not only due to the non-stranded nature of BGI protocol (Fig. 1), but also caused by the difference in RNaseIII and chemical fragmentation methods, a similar phenomenon also identified in SEQC/MAQC III assessment, in which bias in gene and junction detection could still be identified after the effect of anti-sense genes had been removed [12].

RNaseIII specifically recognized RNA secondary structures that contain double-strand and cleaved it into mainly 90 bp fragments (Additional file 4: Figure S4A). We confirmed the cutting pattern (Additional file 4: Figure S4B) reported in a previous study using SOLiD [13], and additionally identified some duplicate reads (Additional file 4: Figure S4B) contained hairpin structure (Additional file 4: Figure S4C).

The read coverage among all transcripts showed no distinctive bias or deflections (Additional file 4: Figure S4D and E), but the coverage within each transcript showed visible differences between RNaseIII and chemical fragmentation (Fig. 5f). Moreover, even the estimated gene expression were highly consistent between the two protocols (Fig. 5a), the coverage of the bases in exon region showed significant differences (Fig. 5g), which suggested that exon-level expression analysis, variation discovery and alternative splicing detection may be affected due to coverage bias between different library construction protocols [45]. Ion Proton sequencing and mapping results demonstrated a similar distribution of genomic categories (Fig. 5g and h) to those by other technologies reported in the ABRF study with poly-A enrichment [11]. However, there is an obvious difference in the proportions identified between using the GENCODE v24 Comprehensive (H) and Basic (I) gene annotations.

## Discussion

With growing interest in applying RNA-Seq for transcriptome annotation, novel transcript discovery, gene expression and other applications, systemic deviations and reproducibility are the two issues that cannot be ignored [13, 46, 47]. Although the influence of library construction methods and sequencing platforms was investigated extensively [12, 13, 45, 48], there has been no dedicated study for Ion Proton RNA-Seq analysis. We have reported a comprehensive assessment of the application of Proton sequencing platform on RNA-Seq, including software and analysis strategies in alignment, gene detection, gene expression quantifications and junction discovery, as well as the bias introduced by different library construction methods.

The phenomenon of increasing length yet deteriorating quality towards the end of a sequencing read is widely accepted and confirmed in platforms like HiSeq, MiSeq, 454 and Ion Proton/PGM [11, 49, 50]. Nevertheless, shorter reads are not necessarily of higher quality, especially when the read length in the same library is not constant. The automatic filter strategy of Torrent Suite trims the 3’ end of reads below certain quality threshold, which can generate short reads passing the quality filter check, but with still lower than average mean read quality. In our study, the highest average read quality is around 120 bp ~ 160 bp (Additional file 2: Figure S2). Ion Proton base calling software derives reads as long as possible, and some long reads with high quality can also be yielded. Therefore, read simulator developers should not only focus on base quality along read position, but should also take the average read quality associated with read length into consideration for Proton.

BWA is an unspliced aligner, widely used in NGS based DNA analysis which align genome resequencing reads back to reference genome. Even it cannot handle long gaps like introns in RNA-Seq data, its good performance in alignment accuracy and efficiency with HiSeq data extends its use in RNA-Seq analysis as SNP and indel calling [1]. The mapping ratio of Proton RNA-Seq data aligned to reference genome by BWA demonstrated strong negative correlation to read length, possibly long reads were more likely to span junction site of multi-exon (Fig. 3), which gives rise to complexities and difficulties in alignment for BWA to open such long gaps for introns [33].

Unspliced aligners TMAP and BWA-SW attained the highest mapping rates against the reference human genome among all programs in this study (Table 2, Additional file 1: Figure S1 and Additional file 2: Figure S2A), and the alignment rates were not affected by read length. For TMAP, reads spanning junction sites or difficult to be mapped were clipped directly at either 3’ or 5’ ends, or mapped poorly at one end with many indels and mismatches. For BWA-SW, reads spanning multi-exon were clipped or aligned multiple times in different exon positions [34]. Compared to TopHat2 that attempts to align each base and does not clip reads at all, TMAP and BWA-SW seem to work in a simpler and more efficient way, though it is not known whether the clipped reads affect the alignment accuracy and contain important information.

Even though the mapping rate is sensitive to parameters, it is still critical to adjust the best combination for Ion Proton data, because some parameters may actually be read length dependent, such as the number of mismatches may increase for longer reads, maximum gap length, maximum edit distance in TopHat2, maximum mismatches in Subread, but the length of read sequenced from Ion Proton is not equal (Additional file 1: Figure S1), which makes it scarcely possible to increase sensitivity without sacrificing specificity.

Moreover, because of the different alignment algorithm and parameters such as mismatch number, error rate or indel penalty among different programs, comparing the mapping ratio directly to evaluate the performance of aligners can hardly be fair [50]. Besides the overall mapping ratio, it is more concerned whether mapping rate is associated with read length. The read length of some other NGS sequencers such as PGM, Roche 454, PacBio RS can also be varying, and likely to be increased by size selection, updating of sequencing enzyme or base calling method. So, how to handle reads with varying length is still challenging in NGS software design.

Our study suggests that the decline of mapping rates of long reads against reference transcriptome by BWA and Bowtie2 was mainly due to the accumulation of sequencing error (Fig. 2c), whilst both sequencing errors and difficulty in junction alignment accounted for the poor mapping performance to genome (Fig. 2d and e). When reference junction annotation was allowed to guide the alignment, it reduced the complexity in read alignment to known junctions, and hence resulted in increased mapping rates (Fig. 2b).

Many gene expression estimation methods relied on alignment results, thus the performance of gene detection and quantification was highly dependent on the alignment rate and accuracy [5]. However, due to the unequal read length and high sequencing error rate, some well-performed software and methods are not suitable in processing Proton RNA-Seq data. RSEM requires strict alignment, which any gaps, indels or clipping of read are not allowed [36]. Considering the sequencing quality and read length with Proton, those criteria can be too harsh. When read length increases, sequencing errors can also accumulate, resulting in extremely low alignment rate for long reads (Table 2, Fig. 2a), which in turn make fewer reads available for gene detection.

The alignment-free Sailfish performed well in error tolerance, were concordant with TaqMan array results and sensitive in gene detection when compared to other methods (Fig. 3, Additional file 3: Figure S3). Besides, the performance of Cuffdiff + TMAP was also remarkable, even better than the official protocol Cuffdiff + TopHat2G [51] (Fig. 3, Additional file 3: Figure S3C and D). This could be due to the high alignment rate of TMAP (Table 2, Fig. 2a). Even though TMAP is an unspliced aligner, which is not designed for mapping RNA-Seq data to a reference genome, because each read can be anchored to a particular exon of a certain gene before exon-exon junctions are detected, unspliced alignment result can also be used to estimate expression level [52]. We also compared the performance of HTSeq, a representative of the count-based methods, with Cuffdiff. HTSeq detected fewer genes than Cuffdiff and the two methods have a similar trend. However, Cuffdiff attained higher consistency with Taqman qPCR results (Additional file 5: Figure S5).

Long Proton reads are more likely to span across (multiple) junctions and thus are more effective in isoform and alternative splicing detection (Fig. 4d) [11]. Nonetheless, they are also more difficult to be aligned against reference genomes. In the presence of a reference annotation guide, the mapping rate of TopHat2 increased significantly (Fig. 2, Table 2), so did the detected junction number (Fig. 4, Additional file 3: Figure S3E). Although Subjunc detected more novel junctions (Fig. 4), they need experimental verification.

In this study, we compared different library construction protocols, initial RNA input, and library insert size, all libraries showed high concordance in gene and junction detection (Additional file 3: Figure S3A and B). We also found that by different fragmentation methods as RNaseIII and chemical, even the overall transcript coverage shown no bias in 3’ or 5’ end (Additional file 4: Figure S4D and E), however the within transcript coverage illustrated different mapping pattern (Fig. 5f), and low concordance in base coverage (Fig. 5g), which indicates that the variation detection and alternative splicing discovery can be affected by the coverage bias introduced during library construction. Therefore the same protocol should be followed in a single project. Finally, although our results demonstrated a comparable distribution of genomic categories (Fig. 5h and i) with the ABRF study result [11], the difference in the proportions identified between using the GENCODE v24 Comprehensive and Basic gene annotations warrants second thoughts before comparison among RNA-seq results. Whether the same software is used to derive genomic profiling, whether the same parameters or definitions of intergenic regions are used, whether the same reference is used are all aspects we should pay attention to before a rigorous conclusion can be drawn.

This is the first study that provides an in-depth RNA-Seq analysis assessment on Proton platform, which facilitates software and method development for sequencing platforms that yields variable sequencing read length and sub-optimal sequencing quality. We also provide a resource of proton data for comparisons with RNA-seq data generated by enriched mRNA using new sequencing platforms, for examples, BGISEQ-500 and Sequel.

## Conclusion

In the study, universal human reference RNA was used for library construction by RNaseIII and an alternative library preparation protocol based on mRNAs chemical fragmentation for Proton sequencing. RNaseIII or chemical fragmentation constructed Proton as well as HiSeq libraries share similar number of gene and junction discovered. We compared a wide spectrum of software developed for analyzing HiSeq data on Proton data by alignment rate, expression level correlation with TaqMan array results, the number of genes detected and junction discovery. Simulated sequencing data were also used to determine the factors that affect alignment rate. K-mer based alignment free quantifier Sailfish was robust in gene quantification and compatible with heterogeneous length and sub-optimal quality reads. With inappropriate mapping programs, long reads, even of high quality, could paradoxically reduce mapping in junctions. Reference guides could partially ameliorate the situation, demonstrated by the superior performance of TopHat2G when compared with TopHat2 in junction alignment. TMAP and BWA-SW manifest high tolerance in sequencing error and unaffected mapping rates for long reads. Decreased alignment rate with longer reads could be due to accumulated sequencing errors, or higher probability of spanning across junctions.

## Method

### Sample preparation

The standard commercial Universal Human Reference RNA (UHRR, 740000, Agilent Technologies) was selected as starting RNA materials because it has been used in benchmarking studies, including MAQCI and SEQC/MAQC-III by US Food and Drug Administration [12, 53]. This sample is composed of total RNA from 10 human cell lines, and the corresponding differential gene expression data measured by Taqman array in MAQCI [53], which is widely used for assessing the accuracy of genes expression quantification in RNA-seq are available [11, 12, 53]. The sample was diluted to 1 μg/μl and the quality was assessed using Agilent Bioanalyzer 2100.

### Ion Proton library preparation and sequencing

#### Library construction based on chemical fragmentation

We used 2 μg or 0.2 μg total RNA (UHRR) as the starting material to enrich mRNA with Dynabeads® mRNA Purification Kit (#61006, Life Technologies) according to the manufacturer’s protocol. The mRNA was then fragmented using 5× first strand buffer and 0.1 ng N6 random primer at 94 °C for 10 min. The first-strand cDNA synthesis was constructed with dNTPs, DTT, RNase Inhibitor and SuperScript® II Reverse Transcriptase (#18064014, Life Technologies). The reverse transcription PCR conditions were as follows: 25 °C for 10 min, 42 °C for 40 min, 70 °C for 15 min, 4 °C hold. The first strand cDNA(ss-cDNA) were incubated with 5× second strand buffer (#10812014, Life Technologies), 20 mMdNTPs, 25U DNA Polymerase I (#P7050L, Enzymatics), 1U RNaseH(Y922L, Enzymatics) at 16 °C for 2 h to synthesize double strand cDNA (ds-cDNA). The ds-cDNA was repaired by T4 Polynucleotide Kinase, T4 DNA polymerase and Klenow fragment with dNTPs to create phosphorylated blunt-end termini. The end-repaired ds-cDNA was then ligated to synthetic A and P adaptors. The adaptor-ligated ds-cDNA was purified with Ampure XP beads (#A63882, Beckman) to remove unincorporated adaptors. The purification libraries were size selected by agarose gel electrophoresis, followed by purification with QIAquick Gel Extraction Kit (#28706, Qiagen). The size selected libraries were inserted with templates of 150 bp ~ 220 bp, and then subjected to PCR (72 °C for 20 min, 95 °C for 5 min, followed by 12 ~ 13 cycles of 95 °C for 30 s, 58 °C for 30 s, 72 °C for 1 min, and kept at 4 °C) in a final 25 μl reaction solution containing 1U Platinum® Pfx DNA Polymerase (#C11708-021, Invitrogen), 1 × Pfx buffer, MgSO4, dNTPs, A primer and P primer. The amplified PCR libraries were purified with Ampure XP beads and eluted in TE buffer.

#### Library construction based on RNaseIII fragmentation

Libraries were prepared from 2 μg of total RNA (UHRR), and mRNA was enriched with Dynabeads® mRNA Purification Kit. The mRNA fragmentation with RNaseIII and following steps were carried out according to Ion Total RNA-Seq Kit v2 recommendation (#4476286E, Life Technologies).

### Sequencing on Ion Proton platform

Emulsion PCR was performed using the One Touch system (Life Technologies). Beads were prepared using the One Touch 2 Template Kit v3 (#4488318). Sequencing was performed by using Ion Proton 200 sequencing kit v3 (#4488315) on the P1 Ion chip. Data were collected using the Torrent Suite v4.0 software.

### Illumina Hiseq2000 library preparation and sequencing

We chose the same amount (2 μg) of UHRR as the starting material to construct HiSeq RNA-Seq libraries for fair comparison. Most library preparation steps were the same as Ion Proton protocol based on chemical fragmentation. The differences lie in that end-repaired ds-cDNA was incubated with Klenow fragment (3´ → 5´exo-) and dATP to create 3’ overhangs, and then was ligated to HiSeq general adaptors. After purifying with Ampure XP beads and size selecting with agarose gel electrophoresis, the insert template was adjusted to 180 bp ~ 250 bp. Finally, the adaptors ligated DNA were amplified with the following conditions: 94 °C for 2 min, and 13 cycles of 94 °C for 15 s, 62 °C for 30 s and 72 °C for 30 s, 72 °C for 10 min in a final volume 25 μl containing 1U Platinum® Pfx DNA Polymerase, 1 × Pfx buffer, MgSO4, dNTPs, and general HiSeq general PCR primers. Sequencing was carried out on HiSeq2000 according to the Illumina protocols for 90 × 2 pair-end sequencing.

### Sequencing data filtering

Since raw reads may contain low quality reads or adaptor sequences, preprocessing before further analysis is necessary. The filtering steps were as follows: adaptor trimming; average quality calculation of the last 15 bases from 3’ end, trimming the end until the average quality is higher than 10; removal of the reads with length less than 30 bp.

### Alignment

To evaluate the performance of software in aligning Ion Proton sequencing data, reads were mapped to reference genome hg38 downloaded from UCSC and reference transcriptome RefSeq v106 downloaded from NCBI (see Data source in Additional file 6: Supplementary materials). Replicate libraries were merged to analyze whether the mapping ratio is associated with read length. Alignment results were visualized by Integrative Genomics Viewer (IGV) [https://www.broadinstitute.org/igv/].

### Read simulation

dwgsim-0.1.11 [54] was used to simulate two sets of RNA-Seq data based on RefSeq transcripts, one of high sequencing quality, with error rate was 0.001, among which 10 % of the errors were indels; and the other of low sequencing quality, with error rate was 0.01, and 80 % of the errors were indels. The length of simulated reads were set to 300 bp with single-end, and then trimmed to 100, 150, 200, 250 bp respectively.

### Gene and isoform expression quantification

Since RefSeq v106 downloaded from NCBI contained predicted transcripts (ID starting with XM or XR), which may affect quantification accuracy, to better understand the performance of gene expression estimated with Ion Proton sequencing platform and library construction methods, we used RefSeq downloaded from UCSC (Data source in Additional file 6: Supplementary materials) in this part, in which only transcripts confirmed by experiment were used. Estimated gene expression level was compared to TaqMan result from MAQC I project [53].

### Base coverage comparison

Isoforms with Fragments Per Kilobase of transcript per Million mapped reads (FPKM) > =10 estimated by TopHat2G + Cuffdiff were selected to avoid random bias due to low expression level and/or coverage. We used SAMtools to calculate the depth of each base in the exon region of these isoforms [54]. Spearman correlation was chosen as the metric to compare the within transcript coverage concordance between libraries constructed by RNaseIII or chemical fragmentation.

### Within-transcript coverage calculation

We used TMAP to align reads to reference transcriptome, and then used SAMtools to calculate the depth of each base for every transcript. To illustrate biases introduced by different library construction methods we compared within-transcript coverage in stably expressed house-keeping genes. R scripts were used in visualizing coverage distribution.

### Read distribution

RSeQC [55] was used to calculate the percentage of reads that map to various gene sequence categories defined in GENCODE v24. When genome features are overlapped, for example, a region can be annotated as exon or intron depending on transcripts, the annotation was processed following the order of: CDS exons > UTR exons > Introns > Intergenic regions specified in RSeQC. Intergenic region was defined as less than 1 kb from transcription start sites (TSS) and transcription end sites (TES), deep intergenic region was defined as between 1 k and 10 K from TSS and TES.

## Abbreviations

NCBI, National Center for Biotechnology Information; qRT-PCR, Quantitative real-time reverse transcription polymerase chain reaction; SEQC, Sequencing Quality Control Consortium; UCSC, University of California, Santa Cruz; UHRR, universal human reference RNA.

## Additional files

Additional file 1: Figure S1: The number of reads by read length in (A) the eleven Proton libraries. The peaks of read length of ProC libraries were around 150 ~ 200 bp, whilst around 90 bp for ProR libraries. (B) Insert sizes of the two HiSeq libraries, calculated by the distance between pair-end reads. (PDF 2520 kb) Additional file 2: Figure S2. Mean read quality distribution. The shade of color (from grey to blue to black) represents the density (percentage) of read with certain mean quality. The reads of highest mean quality were around 120 ~ 160 bp long in ProC libraries, whist for ProR libraries the reads mean quality remained high until 150 bp, where the quality began to deteriorate. (PDF 6020 kb) Additional file 3: Figure S3. (A) Comparison of RNaseIII fragment (ProR), medial insert size (166 bp, ProC_insertM), long insert size (215 bp, ProC_insertL), low initial RNA input (200 ng, ProC_lowinput), and HiSeq with single-end, and pair-end sequencing libraries in gene detection and expression quantification accuracy. Y axis on the left is the number of detected genes, y axis on the right is the Spearman correlation with TaqMan result. All libraries manifest high quantification consistency with TaqMan, pair-end HiSeq library detected 1000 more genes than other libraries. (B) Comparison of libraries in junction detection. All libraries detected more than 120,000 junctions. (C-E) Comparison of detected gene number (C), consistency with TaqMan result (D) and junction discovery (E) by different methods. (PDF 6708 kb) Additional file 4: Figure S4. (A) Length distribution of mRNAs after RNaseIII fragmentation. X axis is fragment length in nt, y axis is the concentration of the fragments. The peak length of both replicates is around 90 nt, (B) Mapping patterns around the mitotic spindle positioning (MISP) gene by the RNaseIII fragmentation (ProR) and chemical fragmentation (ProC) libraries. Mapped reads were visualized by IGV. (C) The secondary structure of a duplicate read of MISP, predicted by RNAfold. The free energy of the thermodynamic ensemble is -21.04 kcal/mol. The frequency of the minimum free energy structure in the ensemble is 11.46 %. The ensemble diversity is 8.87. (D-E) Overall read coverage among transcripts of ProC and ProR respectively. (PDF 6494 kb) Additional file 5: Figure S5. Comparison of gene expression estimate method by HTSeq and CuffDiff in terms of (A) detected gene number, and (B) consistency with TaqMan result. (PDF 2185 kb) Additional file 6: Supplementary Method. The files and commands used in the study. (DOCX 19 kb)
