# Supplementary figures and images for: An optimized protocol for generation and analysis of Ion Proton sequencing reads for RNA-Seq
Source: BMC Genomics. 2016 May 26;17:403. doi: 10.1186/s12864-016-2745-8 (PMC4880854; doi:10.1186/s12864-016-2745-8)

**A**

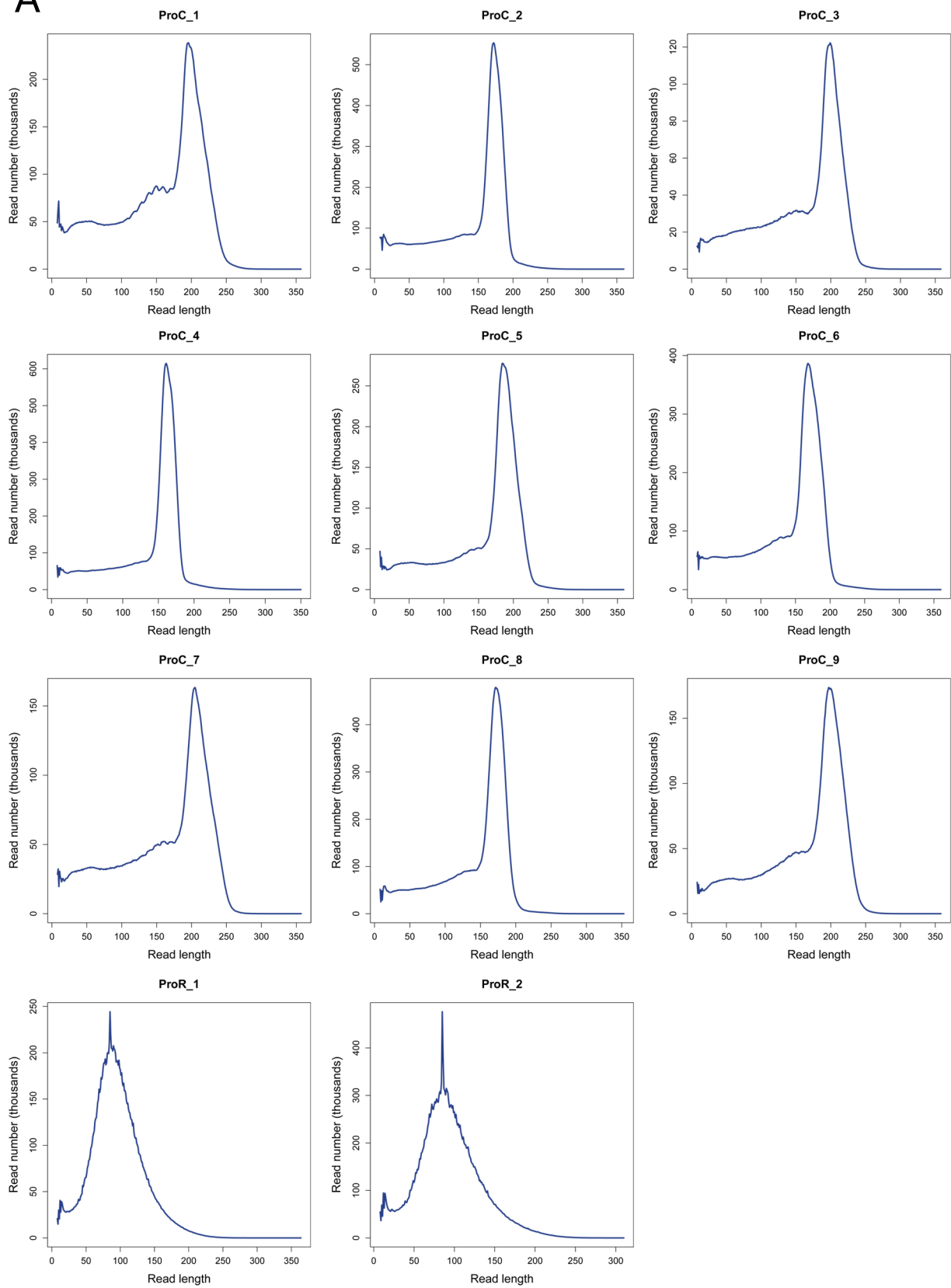

**B**

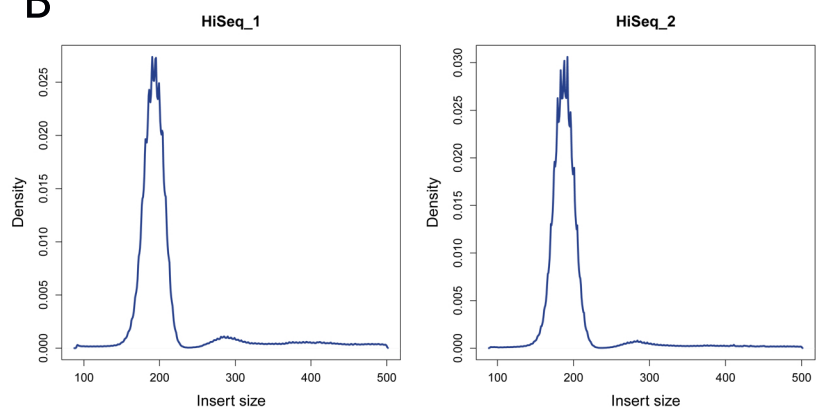

Supplement: Additional file 1: Figure S1: — The number of reads by read length in (A) the eleven Proton libraries. The peaks of read length of ProC libraries were around 150 ~ 200 bp, whilst around 90 bp for ProR libraries. (B) Insert sizes of the two HiSeq libraries, calculated by the distance between pair-end reads. (PDF 2520 kb) [file 12864_2016_2745_MOESM1_ESM.pdf]

ProC\_1

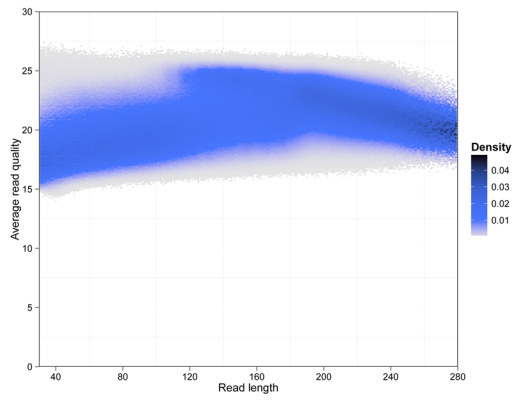

ProC\_2

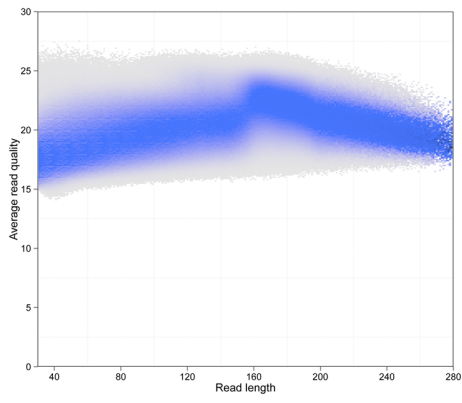

ProC\_3

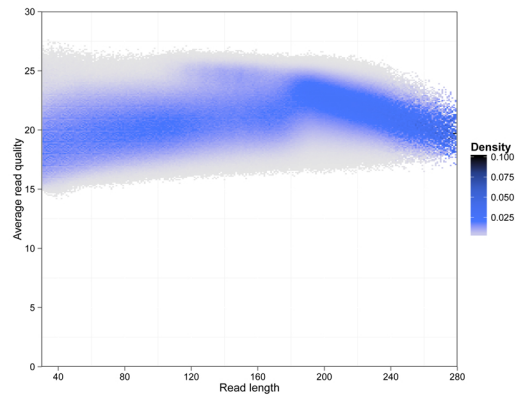

ProC\_4

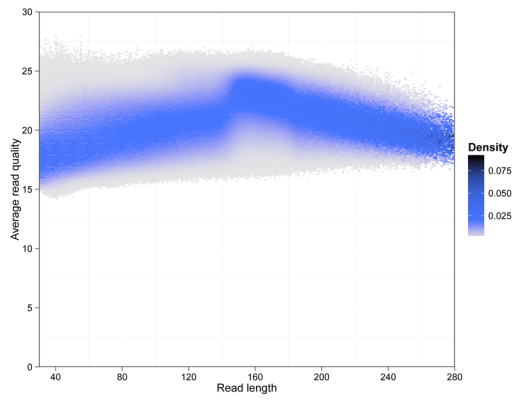

ProC\_5

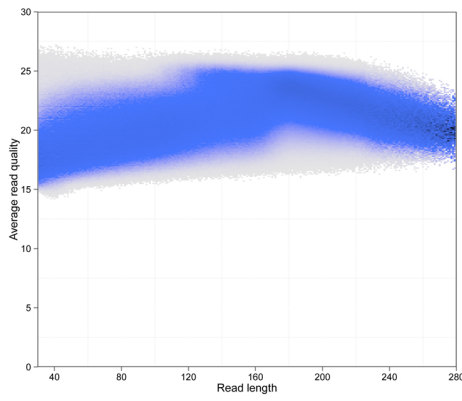

ProC\_6

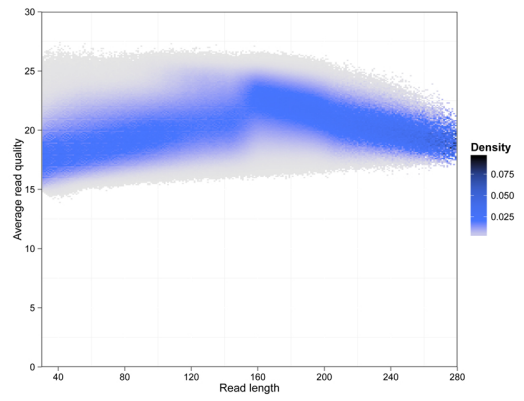

ProC\_7

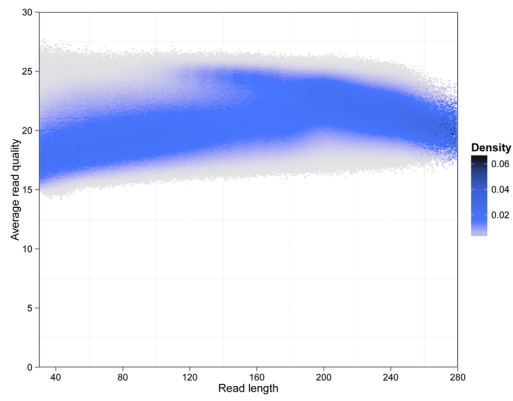

ProC\_8

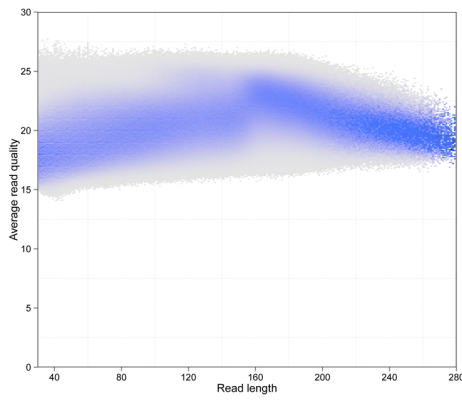

ProC\_9

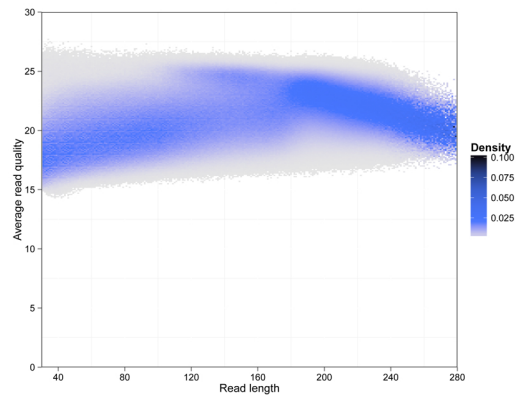

ProR\_1

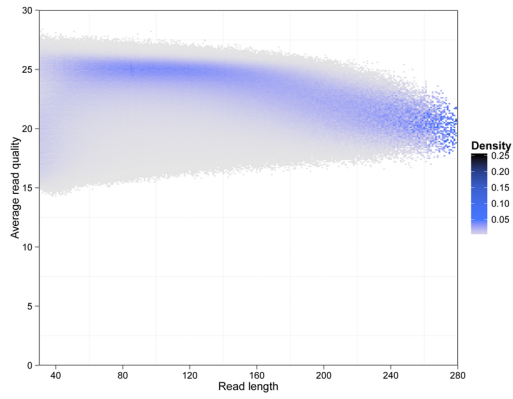

ProR\_2

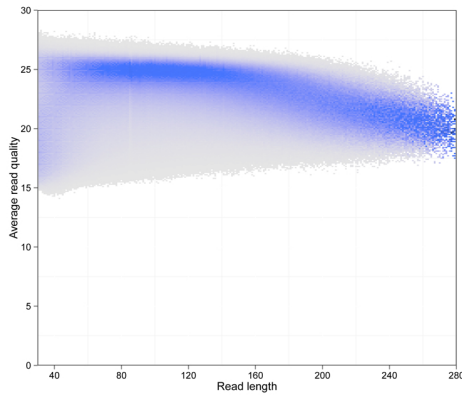

Supplement: Additional file 2: Figure S2. — Mean read quality distribution. The shade of color (from grey to blue to black) represents the density (percentage) of read with certain mean quality. The reads of highest mean quality were around 120 ~ 160 bp long in ProC libraries, whist for ProR libraries the reads mean quality remained high until 150 bp, where the quality began to deteriorate. (PDF 6020 kb) [file 12864_2016_2745_MOESM2_ESM.pdf]

A

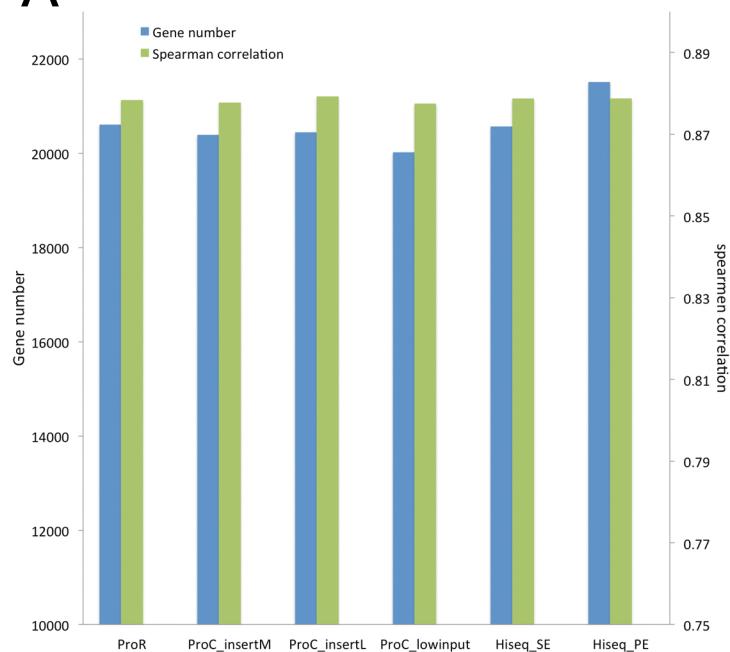

B

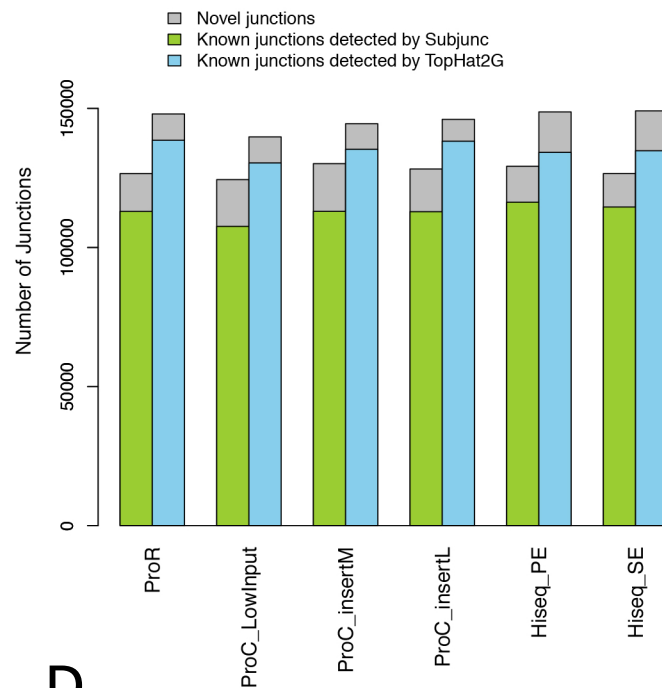

C

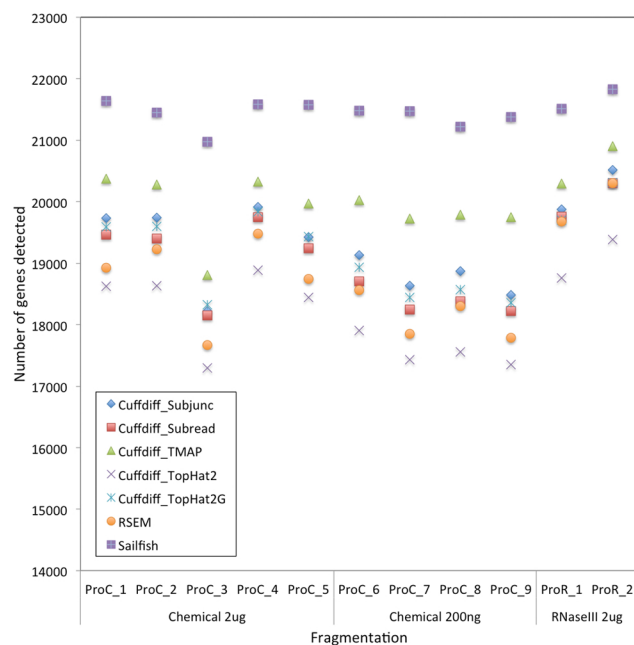

D

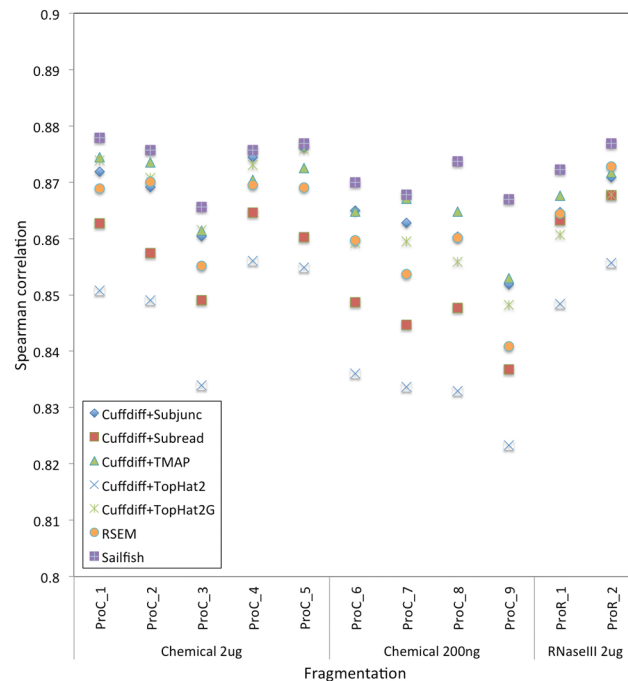

E

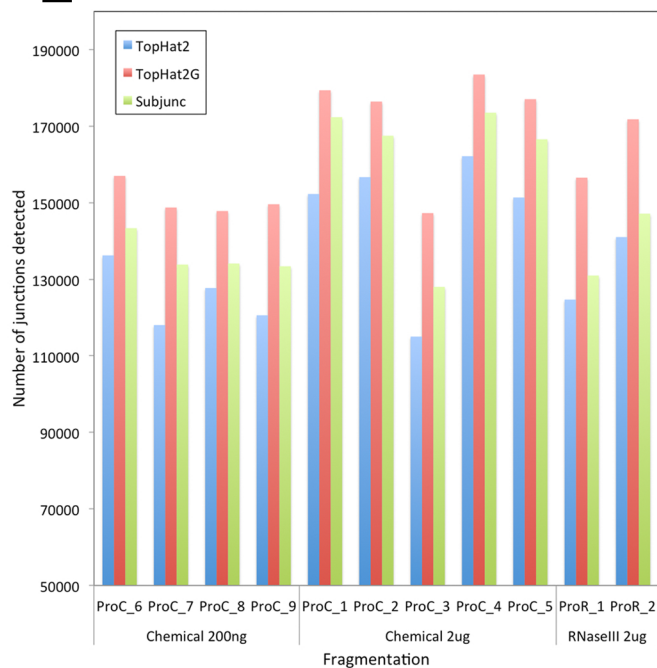

Supplement: Additional file 3: Figure S3. — (A) Comparison of RNaseIII fragment (ProR), medial insert size (166 bp, ProC_insertM), long insert size (215 bp, ProC_insertL), low initial RNA input (200 ng, ProC_lowinput), and HiSeq with single-end, and pair-end sequencing libraries in gene detection and expression quantification accuracy. Y axis on the left is the number of detected genes, y axis on the right is the Spearman correlation with TaqMan result. All libraries manifest high quantification consistency with TaqMan, pair-end HiSeq library detected 1000 more genes than other libraries. (B) Comparison of libraries in junction detection. All libraries detected more than 120,000 junctions. (C-E) Comparison of detected gene number (C), consistency with TaqMan result (D) and junction discovery (E) by different methods. (PDF 6708 kb) [file 12864_2016_2745_MOESM3_ESM.pdf]

A

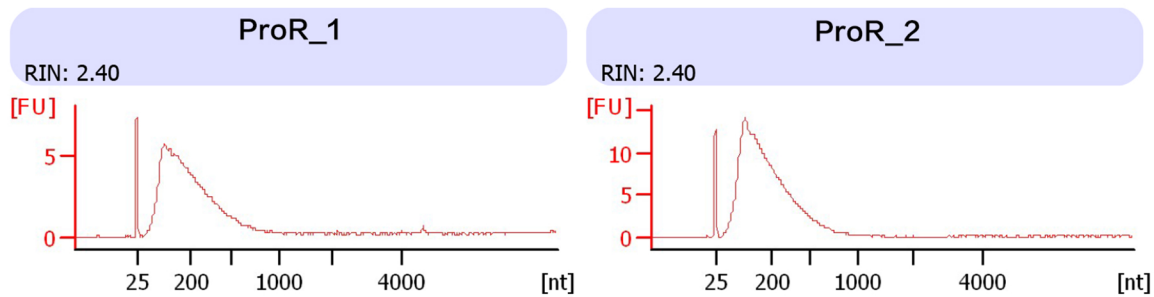

B

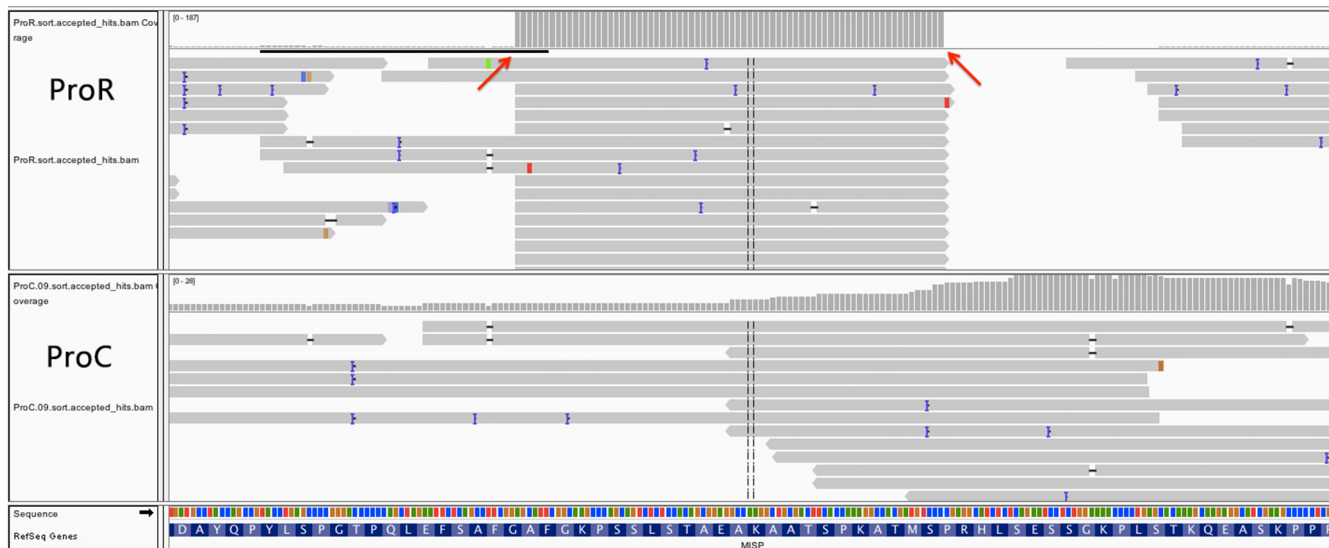

C

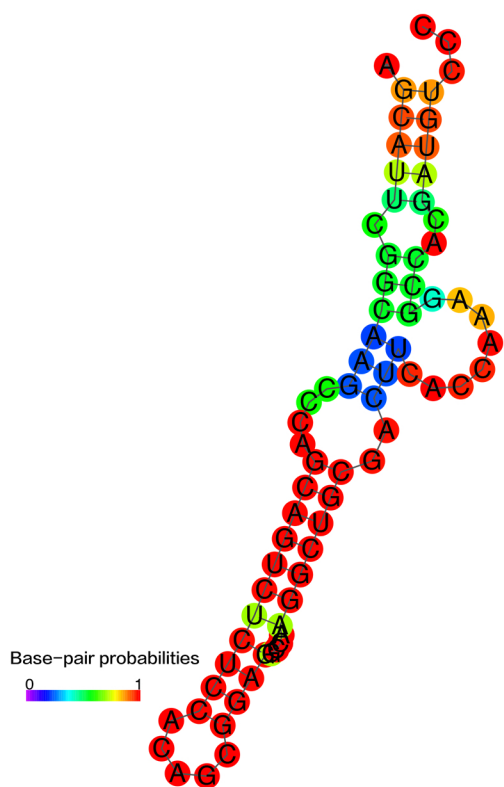

D

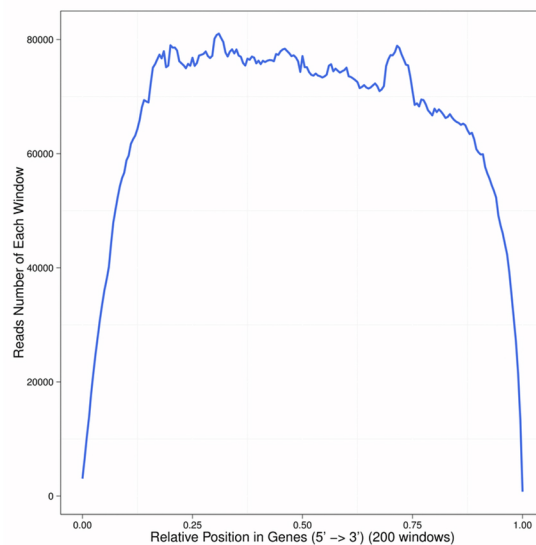

E

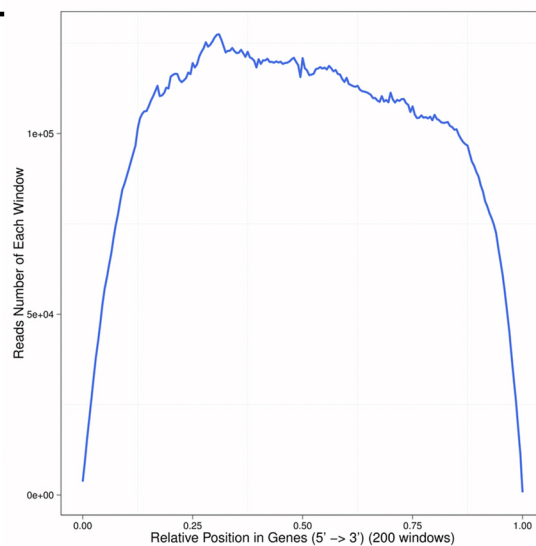

Supplement: Additional file 4: Figure S4. — (A) Length distribution of mRNAs after RNaseIII fragmentation. X axis is fragment length in nt, y axis is the concentration of the fragments. The peak length of both replicates is around 90 nt, (B) Mapping patterns around the mitotic spindle positioning (MISP) gene by the RNaseIII fragmentation (ProR) and chemical fragmentation (ProC) libraries. Mapped reads were visualized by IGV. (C) The secondary structure of a duplicate read of MISP, predicted by RNAfold. The free energy of the thermodynamic ensemble is -21.04 kcal/mol. The frequency of the minimum free energy structure in the ensemble is 11.46 %. The ensemble diversity is 8.87. (D-E) Overall read coverage among transcripts of ProC and ProR respectively. (PDF 6494 kb) [file 12864_2016_2745_MOESM4_ESM.pdf]

A

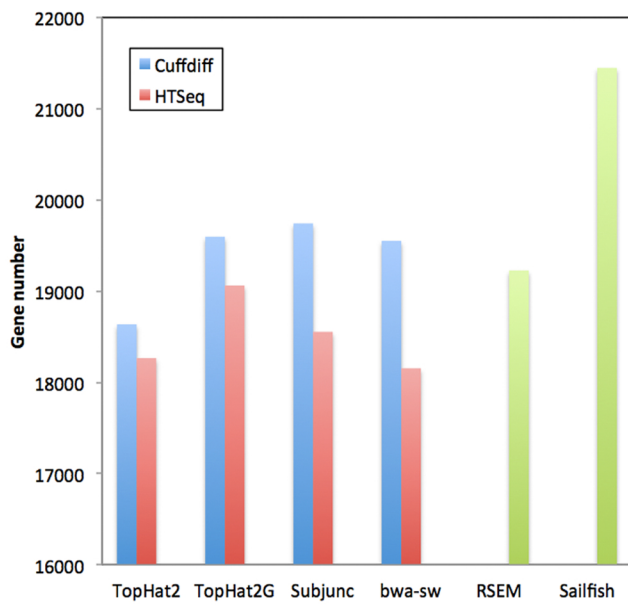

B

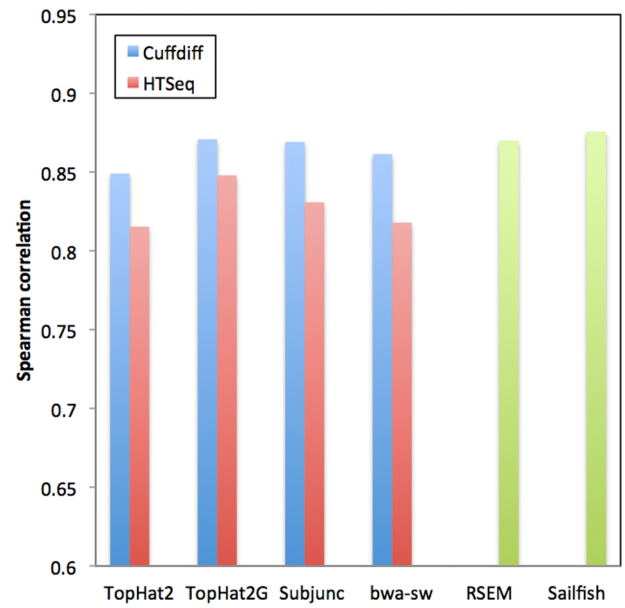

Supplement: Additional file 5: Figure S5. — Comparison of gene expression estimate method by HTSeq and CuffDiff in terms of (A) detected gene number, and (B) consistency with TaqMan result. (PDF 2185 kb) [file 12864_2016_2745_MOESM5_ESM.pdf]
