# Supplementary material for: An optimized protocol for generation and analysis of Ion Proton sequencing reads for RNA-Seq
Source: BMC Genomics. 2016 May 26;17:403. doi: 10.1186/s12864-016-2745-8 (PMC4880854; doi:10.1186/s12864-016-2745-8)
Supplement: Additional file 6: — Supplementary Method. The files and commands used in the study. (DOCX 19 kb) [file 12864_2016_2745_MOESM6_ESM.docx]

This file contains the files and commands used in the study

**Data source:**

In this study, reference genome is hg38, reference transciptome are RefSeq v106, Esemble release-78, Gencode release-21.

**hg38** :

http://hgdownload.soe.ucsc.edu/goldenPath/hg38/bigZips/hg38.fa.gz

**RefSeq v106:**

ftp://ftp.ncbi.nlm.nih.gov/genomes/Homo_sapiens/RNA/rna.fa.gz

ftp://ftp.ncbi.nlm.nih.gov/genomes/Homo_sapiens/GFF/ref_GRCh38_top_level.gff3.gz

**RefSeq (UCSC):**

http://hgdownload.soe.ucsc.edu/goldenPath/hg38/bigZips/refMrna.fa.gz

http://hgdownload.soe.ucsc.edu/goldenPath/hg38/database/refSeqAli.txt.gz

**Ensembl release-78:**

ftp://ftp.ensembl.org/pub/release-78/fasta/homo_sapiens/cdna/Homo_sapiens.GRCh38.cdna.all.fa.gz

**GENCODE release-21:**

ftp://ftp.sanger.ac.uk/pub/gencode/Gencode_human/release_21/gencode.v21.pc_transcripts.fa.gz

**GENCODE release-24:**

Comprehensive gene annotation:

<ftp://ftp.sanger.ac.uk/pub/gencode/Gencode_human/release_24/gencode.v24.annotation.gtf.gz>

Basic gene annotation:

<ftp://ftp.sanger.ac.uk/pub/gencode/Gencode_human/release_24/gencode.v24.basic.annotation.gtf.gz>

**Alignment:**

**Bowtie2**:

Version: bowtie2-2.1.0

default parameters of RSEM (bowtie2 -q --phred33 --sensitive --dpad 0 --gbar 99999999 --mp 1,1 --np 1 --score-min L,0,-0.1 -p 6 -k 200)

**BWA**:

Version: bwa-0.7.10

default

**BWA-SW**: bwa-0.7.10

bwa bwasw -z 3

**Subjunc**:

Version: subread-1.4.6-Linux-x86_64

subread -I 5 -M 10 for Ion Proton; default for HiSeq

**Subread**:

Version: subread-1.4.6-Linux-x86_64

subread -I 5 -M 10

**TMAP**:

Version: 3.4.1

TMAP -a 2 -n 8 -v -Y -u -o 1 stage1 map4

**TopHat2**:

Version: tophat-2.0.8.Linux_x86_64

tophat --b2-very-sensitive --read-gap-length 10 --read-edit-dist 10 --read-mismatches 10 --segment-mismatches 3 --max-insertion-length 5 --max-deletion-length 5 --segment-length 22 -m 2

**TopHat2G**:

Version: tophat-2.0.8.Linux_x86_64

tophat -G mRNA.gtf (providing the reference GTF file) --b2-very-sensitive --read-gap-length 10 --read-edit-dist 10 --read-mismatches 10 --segment-mismatches 3 --max-insertion-length 5 --max-deletion-length 5 --segment-length 22 -m 2

**HISAT:**

Version: hisat-0.1.6-beta

default

**GMAP:**

Version: 2014-08-04

-f samse -n 0

**HTSeq:**

Version: HTSeq-0.6.1p1

Default

**Reads simulation:**

**dwgsim**:

Version: dwgsim-0.1.11

dwgsim –r 0.001 –R 0.1 for high sequencing quality data simulation; –r 0.01 –R 0.8 for low sequencing quality data simulation

**Read distribution**

RSeQC read_distribution.py

Version: RSeQC-2.3.6

default

**Quantification:**

**Cuffdiff**:

Version: cufflinks-2.1.1.Linux_x86_64

default

**Sailfish**:

Version: Sailfish-0.6.3-Linux_x86-64

sailfish –k 18

**RSEM**:

Version: rsem-1.2.12

default

**RNA secondary structure prediction:**

RNAfold WebServer

http://rna.tbi.univie.ac.at/cgi-bin/RNAfold.cgi
